# Narrow bandgap oxide nanoparticles coupled with graphene for high performance mid-infrared photodetection

**DOI:** 10.1038/s41467-018-06776-z

**Published:** 2018-10-16

**Authors:** Xuechao Yu, Yangyang Li, Xiaonan Hu, Daliang Zhang, Ye Tao, Zhixiong Liu, Yongmin He, Md. Azimul Haque, Zheng Liu, Tom Wu, Qi Jie Wang

**Affiliations:** 1 Centre for OptoElectronics and Biophotonics, School of Electrical and Electronic Engineering & The Photonics Institute, Nanyang Technological University, 639798 Singapore, Singapore; 2 Materials Science and Engineering, King Abdullah University of Science and Technology, Thuwal, 23955-6900 Saudi Arabia; 3 Imaging and Characterization Core Lab, King Abdullah University of Science and Technology, Thuwal, 23955-6900 Saudi Arabia; 4 Centre of Programmable Materials, School of Materials Science and Engineering, Nanyang Technological University, 50 Nanyang Avenue, 637371 Singapore, Singapore; 5 School of Materials Science and Engineering, University of New South Wales (UNSW), Sydney, NSW 2052 Australia

## Abstract

The pursuit of optoelectronic devices operating in the mid-infrared regime is driven by both fundamental interests and envisioned applications ranging from imaging, sensing to communications. Despite continued achievements in traditional semiconductors, notorious obstacles such as the complicated growth processes and cryogenic operation preclude the usage of infrared detectors. As an alternative path towards high-performance photodetectors, hybrid semiconductor/graphene structures have been intensively explored. However, the operation bandwidth of such photodetectors has been limited to visible and near-infrared regimes. Here we demonstrate a mid-infrared hybrid photodetector enabled by coupling graphene with a narrow bandgap semiconductor, Ti_2_O_3_ (*E*_g_ = 0.09 eV), which achieves a high responsivity of 300 A W^−1^ in a broadband wavelength range up to 10 µm. The obtained responsivity is about two orders of magnitude higher than that of the commercial mid-infrared photodetectors. Our work opens a route towards achieving high-performance optoelectronics operating in the mid-infrared regime.

## Abstract (web summary)

Coupling graphene with narrow band-gap Ti_2_O_3_ nanoparticles can enable efficient mid-infrared photodetection. Here, the authors report a graphene-Ti_2_O_3_ based hybrid photodetector with high responsivity of ~300 A W^-1^ up to 10 μm by varying the number of graphene layers and size of Ti_2_O_3_ nanoparticles.

## Introduction

High-performance infrared photodetectors that can convert light into electrical signals lead to a wide range of applications ranging from imaging sensors to optical communications^1–3^. Practically, long-wavelength infrared (LWIR) detectors are expected to have features of high photon-carrier conversion efficiency, low noise level at room temperature and fast response speed in a broadband wavelength range. It still remains as a challenge to realize high performance LWIR photodetection with the conventional narrow-bandgap semiconductors such as mercury cadmium telluride (HgCdTe) thin films, indium antimonide (InSb) and artificial semiconductor superlattices via inter/intraband transitions^4^. Recently, significant efforts have been devoted to colloidal quantum dots (CQDs) based photodetectors due to their high absorption efficiency and facile manufacturing processes^5^. However, CQDs-based photodetectors suffer from limited operation spectral bandwidth because of their relatively large bandgaps^6,7^. Alternatively, two-dimensional materials appear to be promising candidates and provide unique properties such as ultrahigh carrier mobility, easy-integrability and broadband light absorption^8^. Graphene has been successfully demonstrated as a broadband photodetector from visible to the terahertz wavelength regimes^9–14^. Unfortunately, the intrinsic low absorption and fast carrier recombination rate of graphene hinder its application in photodetection, and such devices suffer from low responsivity as well as poor detectivity^15^.

Several pioneering works have been devoted to improving the performance of graphene-based photodetectors through constructing hybrid structures. Ultrahigh optical gain was achieved in hybrid graphene-CQD structures, where graphene serves as the ultrahigh speed carrier transport channel and CQD serves as light absorber with high absorption efficiency^16,17^. In such hybrid structures, one type of carrier (either electron or hole) is trapped in the CQD while the other type rapidly circulates in the graphene channel driven by the external source-drain voltage. Similar designs were also employed in the photodetectors with hybrid graphene-TiO_2_^18^, graphene-ZnO^19^, graphene-dye^20^, and graphene-perovskite structures^21^. However, the operation wavelength of such hybrid photodetectors are limited in the visible and the near-infrared regimes owing to the relatively large intrinsic bandgap of the light absorbers^9^. Although black phosphorene^22–25^ and noble metal dichalcogenides have been recently demonstrated as a near-infrared/mid-infrared semiconductor^26^, it still remains as a challenge to further improve the performance of photodetectors in terms of photon absorption efficiency, responsivity, and response speed in the important mid-infrared regime.

Here we demonstrate room-temperature mid-infrared photodetectors with high responsivity, detectivity and high speed, realized by hybridizing graphene with a novel narrow bandgap semiconductor, titanium sesquioxide (Ti_2_O_3_) nanoparticles. Ti_2_O_3_, a stable semiconductor with extraordinary broadband photoresponse characteristics, has a narrow-band gap of 0.09 eV, corresponding to a cut-off absorption wavelength of 13.3 μm^27^. The working mechanism is as follows: when the incident light is absorbed by the Ti_2_O_3_ nanoparticles, the photo-excited electrons are trapped in the Ti_2_O_3_ nanoparticles, while the holes are transferred into the graphene channel, leading to a conspicuous photocurrent measured in a field effect transistor configuration. In addition, we also systematically investigated the photo-generated carrier transfer processes at the interface of graphene and Ti_2_O_3_ in this hybrid photodetector. The developed graphene/Ti_2_O_3_ photodetector is demonstrated to be capable of operating in both accessible atmospheric windows, the mid-wavelength infrared (MWIR: 3–5 μm) and the LWIR (8–12 μm) regimes. The broadband mid-infrared operation bandwidth, high responsivity and detectivity make the graphene/Ti_2_O_3_ a promising candidate for mid-infrared photodetection, providing a novel paradigm for mid-infrared photonics and optoelectronics.

## Results

### Band structure and optical properties of Ti2O3

**Fig. 1:**
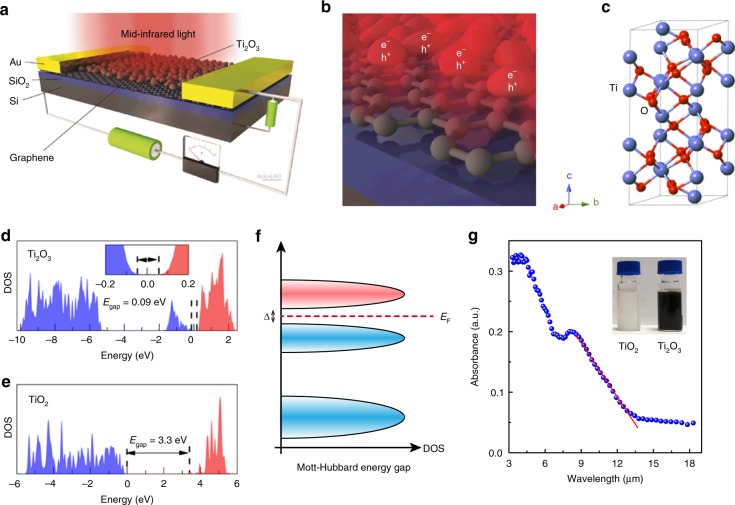
Hybrid graphene/Ti_2_O_3_ nanoparticle mid-infrared photodetector. **a** A schematic diagram of hybrid graphene/Ti_2_O_3_ nanoparticle photodetector. **b** Illustration of the interface between Ti_2_O_3_ nanoparticles and graphene. **c** Crystal structure of corundum Ti_2_O_3_. **d**, **e** Density of states (DOS) of Ti_2_O_3_ and TiO_2_ derived from first principle calculations, respectively. Blue and red areas represent O 2*p* and Ti 3*d* orbitals, respectively. **f** Schematic band diagram of Ti_2_O_3_, indicating that a narrow bandgap is formed between upper Hubbard band (pink) and lower Hubbard band (blue) as a result of strong electron correlation. **g** Absorption spectrum of Ti_2_O_3_ nanoparticles drop-casted on a KBr substrate, measured through Fourier Transform Infrared Spectroscopy. The cut-off wavelength is about 13.3 µm, which corresponds to a band gap of 0.09 eV. The inset is a photo of TiO_2_ and in Ti_2_O_3_ nanoparticles in ether solution

The schematic diagram of the proposed hybrid graphene/Ti_2_O_3_ photodetector is illustrated in Fig. 1a, representing the architecture of the graphene channel decorated with the narrow-bandgap Ti_2_O_3_ nanoparticles as indicated in Supplementary Method. Ti_2_O_3_ exhibits strong light absorption in the infrared regime with wavelength shorter than 13.3 μm, thus it can be used as an efficient light absorber in the MWIR and LWIR regimes. Once electron-hole pairs are mainly generated in the Ti_2_O_3_ nanoparticles with light illumination, the charge transfer process occurs at the graphene/Ti_2_O_3_ interface (Fig. 1b), leading to the conductance modulation of the graphene channel and thus the photodetection. The crystal structure of Ti_2_O_3_ is shown in Fig. 1c, which shows a corundum structure with a trigonal unit cell (*a* = *b* = 5.15 Å; *c* = 13.61 Å). Ti_2_O_3_ is chosen in this hybrid structure as the light absorber owing to its unique narrow bandgap (0.09 eV) that covers the mid-infrared regime^27^. Ti_2_O_3_ is very different from the well-studied wide bandgap semiconductor TiO_2_, as shown in Supplementary Figure 1. To understand the electronic structure and nature of the band edge wavefunction of Ti_2_O_3_, density functional theory (DFT) calculations with a plane-wave basis set were performed. The calculation details are given in the Supplementary Note 1. As shown in Fig. 1d, e, band realignments occur in the density of states (DOS) of Ti_2_O_3_ due to its unpaired 3*d* electrons, which contributes to the states just below the conduction band and forms an upper valence band, as shown in Fig. 1f. The narrow bandgap of Ti_2_O_3_ is located between the lower and the upper Hubbard band^28–31^, which is estimated to be about 0.09 eV according to the absorption spectrum in Fig. 1g. In contrast, the bandgap of TiO_2_ is about 3.3 eV and originated from the separation between O 2*p* and Ti 3*d* orbitals. The unique and wide-band light absorption of Ti_2_O_3_ as shown in Fig. 1f, especially in the mid-infrared range, makes it suitable for creating pertinent mid-infrared optoelectronic devices.

**Fig. 2:**
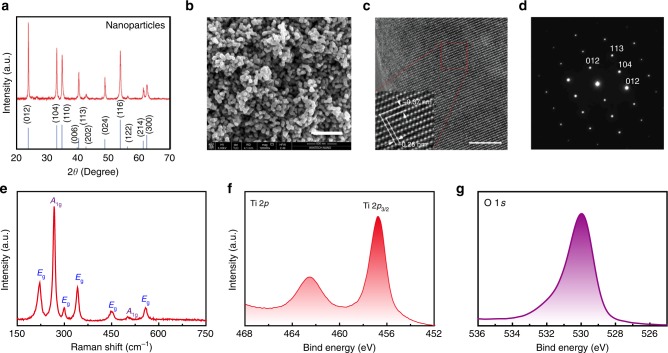
Characterizations of the Ti_2_O_3_ nanoparticles. **a** X-ray diffraction (XRD) pattern of the Ti_2_O_3_ nanoparticles (red) together with the simulated XRD pattern from bulk corundum Ti_2_O_3_ (blue). **b** Scanning electron microscope (SEM) image of the Ti_2_O_3_ nanoparticles. Scale bar: 500 nm. **c** High resolution transmission electron microscope (HR-TEM) image of the Ti_2_O_3_ nanoparticles. Scale bar: 5 nm. Inset shows the enlarged lattice. **d** Corresponding selected area diffraction (SAED) pattern of the Ti_2_O_3_ nanoparticles. **e** Raman spectrum of the Ti_2_O_3_ nanoparticles. The seven characteristic peaks are indicated as 2*A*_1g_+5*E*_g_. **f**, **g** X-ray photoelectron spectroscopy (XPS) of the Ti_2_O_3_ nanoparticles, depicting the Ti 2*p* and O 1*s* peaks, respectively

Ti_2_O_3_ nanoparticles with relatively uniform nanoparticle sizes were prepared by a simple ball milling process followed by ultrasound process and purification in ether solution. Then they were spin-coated on the channel surface of a graphene field effect transistor (FET). As shown in the X-ray diffraction (XRD) pattern in Fig. 2a, the Ti_2_O_3_ nanoparticles exhibit the corundum phase, which is consistent with the previous reports^27,31^. As shown in Fig. 2b and the Supplementary Figure 2, the obtained Ti_2_O_3_ nanoparticles have an average diameter of ~50–200 nm, characterized by scanning electron microscopy (SEM). Furthermore, high-resolution transmission electron microscopy (HR-TEM) and selected-area electron diffraction (SAED) were performed to characterize the structure of the fabricated Ti_2_O_3_ nanoparticles. As shown in Fig. 2c, the lattice constants of ~0.37 nm and ~0.26 nm correspond to the (012) and (104) planes, respectively, in the corundum (trigonal) Ti_2_O_3_ lattice. Moreover, the corresponding SAED pattern shown in Fig. 2d demonstrates the high crystalline quality of the Ti_2_O_3_ nanoparticles. The Raman spectrum of the Ti_2_O_3_ nanoparticles shows seven active Raman modes (Fig. 2e), which is characteristic of corundum Ti_2_O_3_ (space group: R-3c). X-ray photoelectron spectroscopy (XPS) was used to characterize the electronic states of Ti and O in the Ti_2_O_3_ nanoparticles. As shown in Fig. 2f, the Ti 2*p*_3/2_ peaks with binding energies of 456.8 and 462.5 eV arise from spin-orbit splitting, corresponding to Ti^3+^ in Ti_2_O_3_^32^. In addition, the peak with a binding energy of 529.6 eV in the O 1*s* spectrum (Fig. 2g) is consistent with the electronic state of oxygen in Ti_2_O_3_^33^.

### Charge transfer processes in hybrid graphene/Ti_2_O_3_

**Fig. 3:**
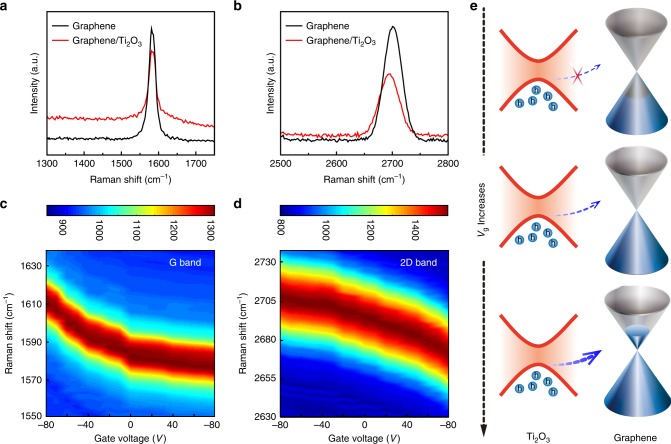
Charge transfer and band alignment in the hybrid graphene/Ti_2_O_3_ structure. **a** G-peaks (~1580 cm^−1^) of the Raman spectra measured on the hybrid graphene/Ti_2_O_3_ and the reference graphene samples without gating voltage. **b** 2D-peaks (~2700 cm^−1^) of the Raman spectra measured on the same samples without gating voltage. **c**, **d** Evolution of the G and 2D Raman peaks, respectively, of the hybrid graphene/Ti_2_O_3_ sample under different gate voltages. **e** Illustration of the band alignment and the charge transfer at the hybrid graphene/Ti_2_O_3_ interface. It is illustrated that holes can only transfer from p-type Ti_2_O_3_ to the graphene layer when the graphene is gated properly. Gray and lightblue regimes represent conduction and valance band, respectively

To examine the interaction between graphene and Ti_2_O_3_ in the hybrid structure, Raman spectroscopy was applied to monitor the charge transfer between Ti_2_O_3_ and graphene under laser illumination. The positions of the characteristic Raman peaks such as the G peak and 2D peak of graphene are sensitive to the carrier density which can be modulated by doping and external electrical bias^34,35^. Hereafter, if not especially declared, graphene refers to monolayer graphene. The doping effect in graphene caused by the charge transfer is investigated by examining the shifts of the G peak and 2D peak. As shown in Fig. 3a, b, both the G peak and the 2D peak exhibit weak redshift in the Raman spectra after the deposition of Ti_2_O_3_ on top of graphene channel. The redshift of G peak indicates the *p*-type doping effect induced by the charge transfer (holes) from Ti_2_O_3_ to graphene in the hybrid structure^34^. Notably, as fabricated Ti_2_O_3_ is a *p*-type semiconductor^36^, and our fabricated pristine graphene is also *p*-type due to the absorbed hydrocarbon molecules or humidity. As a result, the charge transfer in this hybrid structure remains inefficient because of the band misalignment between graphene and Ti_2_O_3_, which contributes to the weak shift of the G peak shown in Fig. 3a.

We thus applied gate voltages to modulate the Fermi level of graphene and align the band structure between graphene and Ti_2_O_3_ to investigate the charge transfer process in the hybrid structure. Figure 3c, d display the gate-dependence of the G peak and 2D peak positions. Clearly, both G peak and 2D peak show clear redshift when the gate voltage changes from −80 to 80 V, which are more significant than the unbiased cases (as shown in Fig. 3a, b). The shift of these Raman peaks provides paramount insights into the charge transfer at the graphene/Ti_2_O_3_ interface^37–39^. The 2D peak is derived from a second-order, double-resonant Raman scattering mechanism and its frequency decreases with increasing gate voltage that up-shifts the Fermi level of graphene^34^. Theoretically, it is proven that with the increase of gate voltage, the G peak which is originated from the coupling between G-phonon lattice vibrations and Dirac fermions should present first blue-shift in the *p*-type doping regime (*V*_g_ < *V*_D_) for graphene and then red-shift in the *n*-type doping regime (*V*_g_ > *V*_D_) for graphene^38^. This is contrary to the experimentally observed trend of G peak shown in Fig. 3c. We attribute the evolution of the G peak to the following two coexisting effects: the gating effect from the external bias and the charge transfer effect at the graphene/Ti_2_O_3_ interface. In the *p*-type doped regime of graphene (*V*_g_ < *V*_D_), the gating effect on graphene is the dominant contribution to the red-shifts of both G peak and 2D peak as the charge transfer process is suppressed, as shown in Fig. 3e. However, the hole transfer from Ti_2_O_3_ to graphene channel begins to dominate in the *n*-type doped regime of graphene (*V*_g_ > *V*_D_) when the Fermi level is upshifted.

### Mid-infrared photodetection under photoconductivity regime

**Fig. 4:**
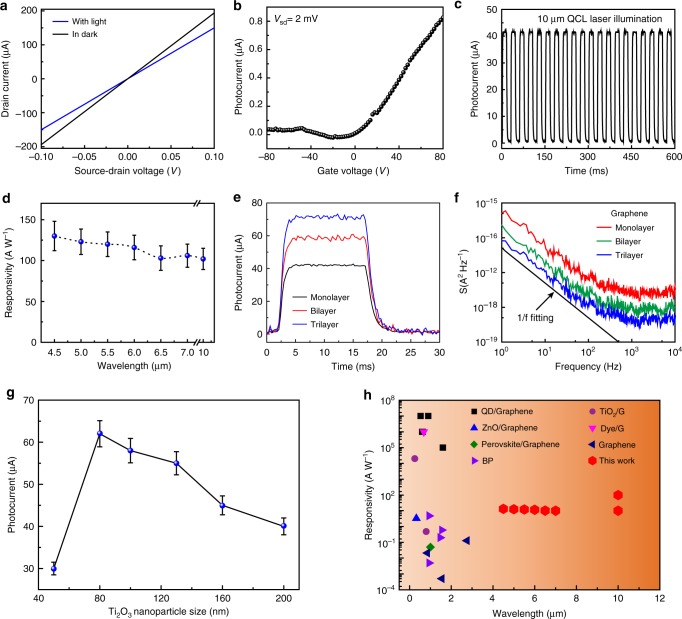
Performance of the hybrid graphene/Ti_2_O_3_ photodetector. **a** *I*_d_ − *V*_d_ curves of the hybrid graphene/Ti_2_O_3_ photodetector measured in dark and under quantum cascade laser illumination (*λ*: 10 µm; power: 2 mW/cm^2^); the gate voltage is 80 V. The channel size is 10.8 µm × 19.5 µm. **b** Corresponding photocurrent of the hybrid graphene/Ti_2_O_3_ photodetector plotted as a function of bottom gate voltage. **c** Temporal response of the device to the illumination of 10 μm laser. **d** Responsivity of the hybrid graphene/Ti_2_O_3_ photodetector measured at different illumination wavelengths in the mid-infrared regime. The high responsivity measured in the wavelength regime from 4.5 to 10 μm demonstrated the broadband operation of the hybrid graphene/Ti_2_O_3_ photodetector. Error bars represent standard deviation. **e** Time-dependent photocurrent of the hybrid graphene/Ti_2_O_3_ photodetector with monolayer, bilayer and trilayer graphene. In the measurements of (**c**) and (**e**), the laser power was kept at 2 mW/cm^2^, and the source-drain voltage (*V*_d_) and gate voltage (*V*_g_) were kept at 2 mV and 80 V, respectively. **f** Noise spectra of monolayer, bilayer and trilayer graphene hybrid photodetectors as a function of frequency measured at a constant bias voltage of 5 mV. All the current noises show a typical trend of S~1/f^α^ with *α* = 1. **g** Photocurrents of hybrid graphene/Ti_2_O_3_ photodetectors with different sizes of Ti_2_O_3_ nanoparticles measured under 10 µm laser illumination. The measurement parameters are the same as in Fig. 4c, d. Error bars represent standard deviation. **h** Comparison of the performance of the hybrid graphene/Ti_2_O_3_ photodetector with the state-of-the-art

With the strong optical absorption of the Ti_2_O_3_ nanoparticles in the mid-infrared regime and the efficient charge transfer in the hybrid graphene/Ti_2_O_3_ structure modulated by proper gate voltage, the mid-infrared photodetection performance of such hybrid photodetectors was demonstrated. The fabrication details of the photodetector can be found in the Supplementary Method. Photodetection measurements were performed first under illumination by two quantum cascade lasers (QCL) with tunable wavelengths from 4 µm to 7 µm and 9.6 µm to 10.6 µm, respectively, as indicated in Supplementary Note 2. As shown in Fig. 4a, b, electrical characterization is recorded both in dark and under the 10 µm laser illumination. The *I*_d_−*V*_d_ curves are generally linear and symmetric for small source-drain voltages, indicating ohmic-like electrode contacts. An obvious decrease of channel current is observed with laser irradiation, which is contributed by the photocurrent as shown in Fig. 4c. The gate-dependent photocurrent of the hybrid graphene/Ti_2_O_3_ structure, which is defined as the absolute value of the source-drain current under the laser illumination subtracted by the dark current, is shown in Fig. 4b. The net photocurrent is almost negligible for gate voltage lower than the Dirac voltage (note that the pristine graphene as fabricated in our experiments is *p*-doped), which is consistent with the inefficient charge transfer due to the band misalignment between graphene and Ti_2_O_3_ in the hybrid structure, as shown in Fig. 3e. In contrast, the photocurrent increases when the gate voltage is higher than the Dirac voltage, indicating that the photogenerated holes are efficiently injected from Ti_2_O_3_ into graphene.

The hybrid graphene/Ti_2_O_3_ photodetector shows a broadband photoresponse in the mid-infrared regime as shown in Fig. 4d and Supplementary Figure 5. As a demonstration example, a net photocurrent of ~ 50 µA, corresponding to a responsivity of ~ 120 A W^−1^ under a source-drain bias of 2 mV, was achieved with 10 μm laser illumination. This indicates that our hybrid photodetector offers low electrical power consumption. The obtained responsivity here vastly surpasses those in the previous reports on graphene- and other 2D-based mid-infrared photodetectors^40–43^, which is about two orders of magnitude higher than that of commercial mid-infrared photodetectors based on MCT, InSb and so on^4,44–46^. Figure 4e exhibits the photoresponse measured in one on/off period with the subtraction of the dark current background. In addition, the response time is determined by the rising (falling) time from 10–90% (90–10%) of the photocurrent in Fig. 4e and Supplementary Figure 8. The rising and falling time was estimated to be ~ 1.2 ms and ~ 2.6 ms, respectively. In the demonstrated photodetector, the response speed is limited by the scattering/trapping centers at the graphene/Ti_2_O_3_ interface introduced during the device fabrication process^47^. The device also demonstrated good stability during the on/off measurements, and the performance remains stable after several weeks if the device is properly kept. Furthermore, the hybrid graphene/Ti_2_O_3_ photodetector shows versatile photoresponse in a broad spectrum range from 4.5 µm to 10 µm as shown in Fig. 4d and the Supplementary Figure 5, which is limited by the tuning range of our laser sources.

## Discussion

The photoresponse of the hybrid graphene/Ti_2_O_3_ photodetector presents a noteworthy enhancement when the graphene channel increases from monolayer to trilayer as shown in Fig. 4e. The electrical properties of the graphene FETs are indicated in Supplementary Figures 6–8. The layer-dependent charge transfer efficiency accounts for the competition between screening and absorption of the electric field of the dipoles of semiconducting Ti_2_O_3_ nanoparticles and the graphene layer as explained in Supplementary Figure 9 and Supplementary Note 3. We achieve an optimized responsivity of ~ 300 A W^−1^ for hybrid trilayer graphene/Ti_2_O_3_ photodetector under 10 µm laser illumination. Another prominent feature of the hybrid photodetector is its high detectivity as obtained from the low noise spectrum as shown in Fig. 4f. The spectral noise in the dark characterized by the setup as shown in Supplementary Figure 4 exhibits a concrete 1/f component, which is consistent with the previous reports for graphene-based FETs^48^. The detectives, D*, are calculated to be ~2 × 10^8^ cm Hz^1/2^ W^−1^, 3 × 10^8^ cm Hz^1/2^ W^−1^, and 7 × 10^8^ cm Hz^1/2^ W^−1^ for monolayer, bilayer and trilayer graphene according to Supplementary Equation 1, respectively, which are comparable with the current room-temperature operated MCT and bolometers^43–45^. Graphene layer dependent photoresponse properties are shown in Supplementary Note 3. This concrete phenomenon additionally demonstrates the surface-mediated charge transfer effect of the abnormal behavior of this hybrid structure^46^. The results here provide hints towards optimizing the 0D-2D hybrid infrared optoelectronic devices.

We further explored the effect of the size of Ti_2_O_3_ nanoparticles on the optoelectronic properties of the hybrid graphene/Ti_2_O_3_ structure. As shown in Fig. 4g, the photocurrent increases with the decrease of the average size of Ti_2_O_3_ nanoparticles from 200 to 80 nm (the size is characterized by TEM as shown in Supplementary Figure 3); however, the photocurrent almost vanishes when the size drops to 50 nm as shown in Supplementary Figure 10. The effect on the size-dependent photocurrent can be attributed to the trade-off between charge transfer efficiency and light absorption efficiency, as explained in Supplementary Note 4. On the other hand, Ti_2_O_3_ thin films deposited on graphene layer by PLD method are characterized by Raman spectrum (Supplementary Figure 11) and patterned into FET channel as shown in Supplementary Figure 12. The photoresponses of the Ti_2_O_3_ thin films based photodetector is also indicated in Supplementary Figure 13 and Supplementary Note 5. The exotic properties of the hybrid graphene/Ti_2_O_3_ make it a promising candidate for mid-infrared photodetection. Moreover, as shown in Fig. 4h, compared with previous reports on similar hybrid photodetectors, the performance of our hybrid graphene/Ti_2_O_3_ photodetector is superior to other systems such as pure graphene^11–14^, black phosphorene^22–25^ and other similar semiconductor hybrid structures. By coupling graphene with narrow-bandgap Ti_2_O_3_, such a hybrid photodetector has the advantages of operating at LWIR with high responsivity and speed. Possible strategy to improve the performance of such hybrid graphene/Ti_2_O_3_ photodetectors is to further enhance the charge transfer rate and efficiency by engineering the surface states of graphene and Ti_2_O_3_ nanoparticles with chemical treatments or by introducing suitable ligands on the surfaces of Ti_2_O_3_ nanoparticles.

To summarize, room-temperature-operated photodetectors with high photoresponse in the mid-infrared regime were demonstrated using the hybrid graphene/Ti_2_O_3_ structures. This type of hybrid photodetector exhibits a high responsivity that surpasses other two-dimensional materials based photodetectors and commercial photodetectors in the long-wavelength infrared range. The unparalleled performance demonstrated in this work hinges upon the highly efficient broadband absorption of Ti_2_O_3_ nanoparticles, as well as the fast carrier transport in graphene under the bias-optimized band alignment conditions. We also elucidate that the charge transfer efficiency increases with the number of graphene layers and achieves a high responsivity of ~ 300 A W^−1^ and detectivity of ~ 7 × 10^8^ cm Hz^1/2^ W^−1^ at room temperature in the hybrid Ti_2_O_3_ device with trilayer graphene hybrid photodetectors. This work not only highlights the importance of exploring narrow-bandgap absorbers and tailoring the charge transfer process for hybrid graphene photodetectors but also opens a venue for developing graphene-based optoelectronic devices for operation in the mid-infrared regime.

## Methods

### Fabrication of the Ti_2_O_3_ nanocrystals

Ti_2_O_3_ raw material was purchased from Sigma-Aldrich. Millipore filter membranes were purchased from Merck Millipore. Ti_2_O_3_ nanoparticles were obtained by using ball milling technique with Agate balls (diameter: 10 and 5 mm). Ethanol was added during milling, and the milling speed was fixed at 300 rpm for 48 h. The size distribution of the obtained nanoparticles is shown in the Supplementary Figure 2. Different sizes of nanoparticles were achieved by tuning the centrifugation speed, as shown in the Supplementary Figure 3.

### Light absorption measurements

The absorption spectra of the Ti_2_O_3_ nanoparticles spin-coated on KBr substrate were measured using a Fourier Transform Infrared Spectrometer (FTIR, VERTEX 70v). The measurements were all performed in the ambient conditions.

### FET device fabrication

Graphene flakes were mechanically exfoliated from a crystal of highly oriented pyrolytic graphite (HOPG) using adhesive 3M-tape and deposited on a silicon wafer with a 285-nm thermalized SiO_2_ layer. The location and quality of graphene were characterized by optical contrast using an optical microscope and Raman spectroscopy, respectively. Then, graphene-based FETs with the heavily doped silicon substrate as the backgate electrode were fabricated by standard photolithography and e-beam evaporation. Subsequently, the Ti_2_O_3_ nanoparticles were dispersed in ethanol solution (~5%, wt %) and spin-coated (4000 rpm) on the surface of the graphene channel. The devices were then dried in an oven under 100 °C for 12 h.

### Electrical and optoelectronic measurements

The electrical characteristics were examined by a semiconductor parameter analyzer (Agilent, B1500A). The responsivity measurement was performed in a digital deep level transient spectroscopy (BIORAD) system with mid-infrared lasers (Tunable CW/Pulsed External Cavity Quantum Cascade Laser from 4.0 µm to 7 µm and 9.75 µm to 10.48 µm) to illuminate the whole devices, as shown in Supplementary Figure 4. The lasers beams are focused by IR lens with a spot size of ~100 μm, which is much larger than the active area of the devices. Noise spectra were acquired by a spectrum analyzer (Keysight M9018A). The measurement details are shown in Supplementary Note 2.

## Electronic supplementary material

Supplementary Information

## Data Availability

The authors declare that the data supporting the findings of this study are available within the paper and its supplementary information files.
