# Supplementary material for: Narrow bandgap oxide nanoparticles coupled with graphene for high performance mid-infrared photodetection
Source: Nat Commun. 2018 Oct 16;9:4299. doi: 10.1038/s41467-018-06776-z (PMC6191432; doi:10.1038/s41467-018-06776-z)
Supplement: Supplementary file 1 — Supplementary Information [file 41467_2018_6776_MOESM1_ESM.pdf]

Supplementary Information

Narrow bandgap oxide nanoparticles coupled with graphene for high-performance mid infrared photodetection

Xuechao Yu,[1,] † Yangyang Li,[2,] † Xiaonan Hu,[1] Daliang Zhang,[3] Ye Tao,[1] Zhixiong Liu,[2] Yongmin He,[1] Md. Azimul Haque,[2] Zheng Liu,[4] Tom Wu,[2,5,*] Qi Jie Wang[1,*]

[1]Centre for OptoElectronics and Biophotonics, School of Electrical and Electronic Engineering & The Photonics Institute, Nanyang Technological University, 639798, Singapore

[2]Materials Science and Engineering, King Abdullah University of Science and Technology, Thuwal 23955-6900, Kingdom of Saudi Arabia

[3]Imaging and Characterization Core Lab, King Abdullah University of Science and Technology, Thuwal 23955-6900, Kingdom of Saudi Arabia

[4] Centre of Programmable Materials, School of Materials Science and Engineering, Nanyang Technological University, 50 Nanyang Avenue, 637371, Singapore

[5] School of Materials Science and Engineering, University of New South Wales (UNSW), Sydney, NSW 2052, Australia

*Corresponding author: tom.wu@unsw.edu.au (T.W.); qjwang@ntu.edu.sg (Q.J.W.)

Supplementary Figures

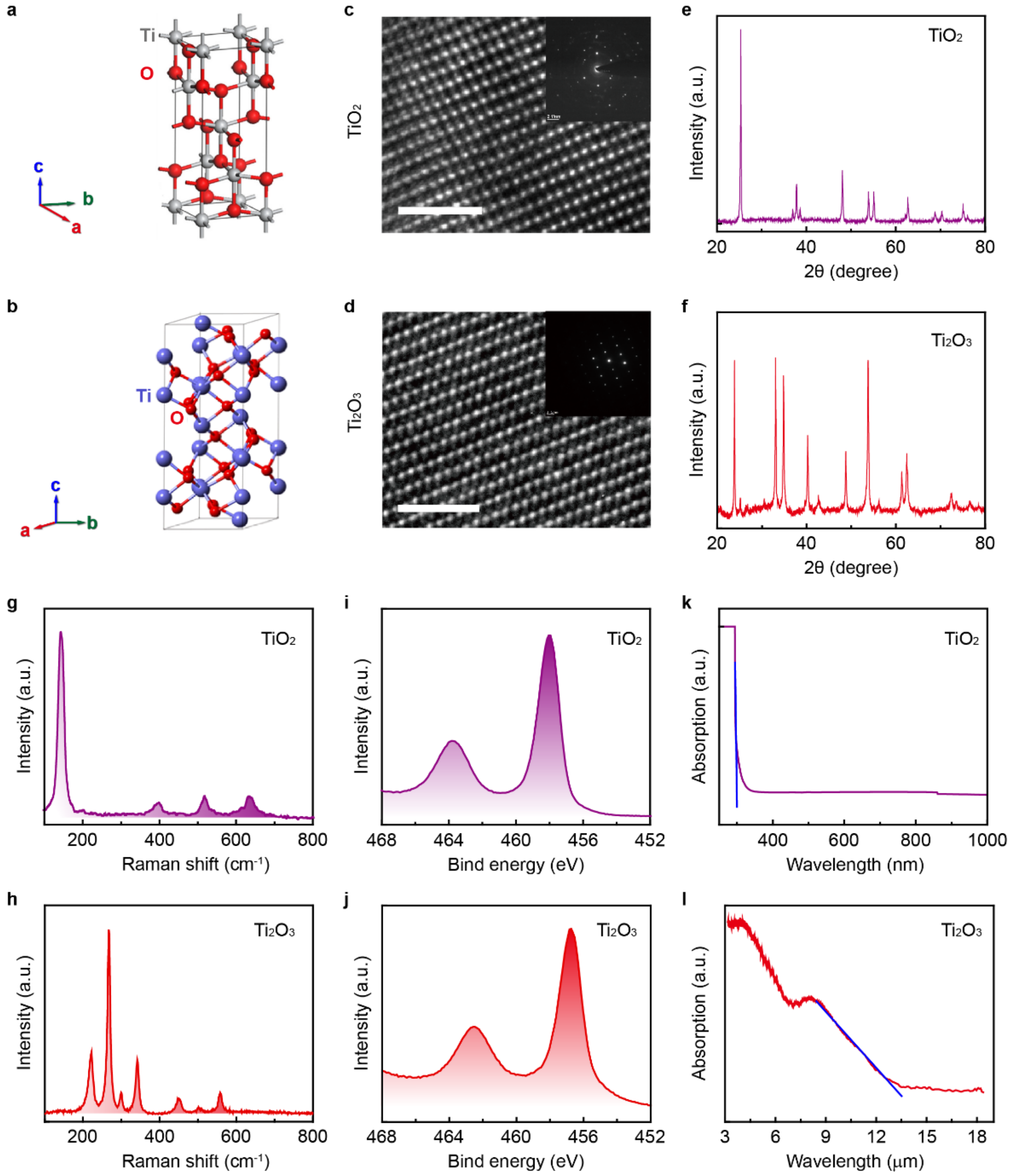

Supplementary Figure 1. Comparison of TiO_2 and Ti_2O_3. (a,b) Crystal structures of anatase TiO_2 and corundum Ti_2O_3. (c,d) HRTEM images and corresponding selected area diffraction (SEAD) patterns of anatase TiO_2 and corundum Ti_2O_3. (e,f) X-ray diffraction (XRD) patterns of anatase TiO_2 and corundum Ti_2O_3. (g,h) Raman spectrum of anatase TiO_2 and corundum Ti_2O_3. (i,j) X-ray photoelectron spectroscopies (XPS) of anatase TiO_2 and corundum Ti_2O_3. (k,l) Absorption spectrum of anatase TiO_2 and corundum Ti_2O_3.

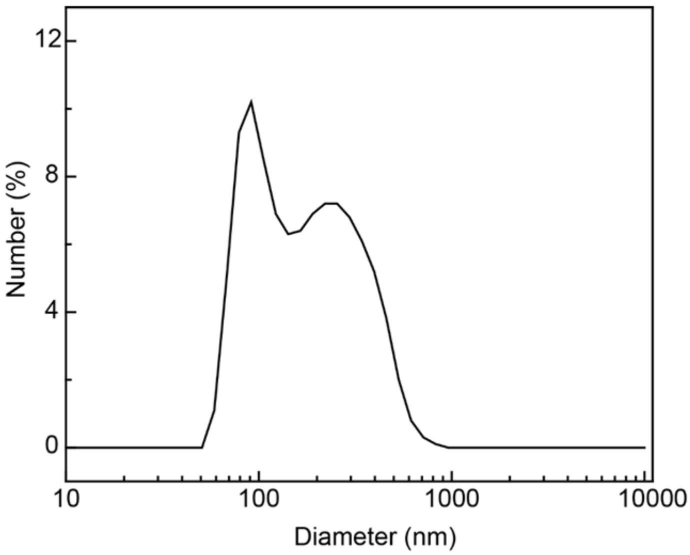

Supplementary Figure 2. Size distribution of the Ti_2O_3 nanoparticles in ethanol solutions. The average size is ~ 200 nm.

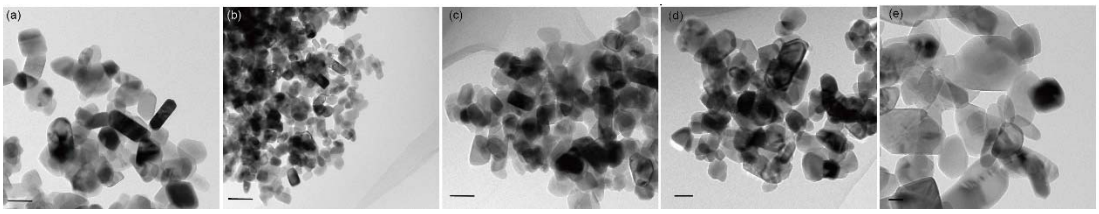

Supplementary Figure 3. TEM images of Ti_2O_3 nanoparticles with different sizes. The scar bar in (a) is 50 nm, the scale bares in b-e are 100 nm.

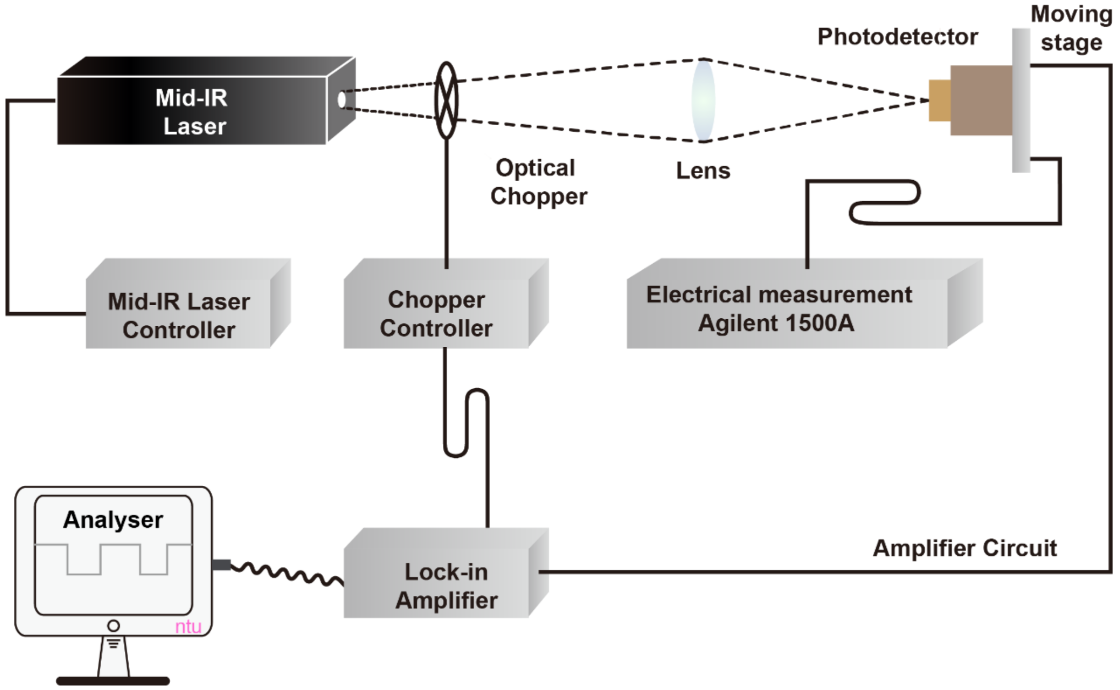

Supplementary Figure 4. Experimental setup of the photodetection measurement.

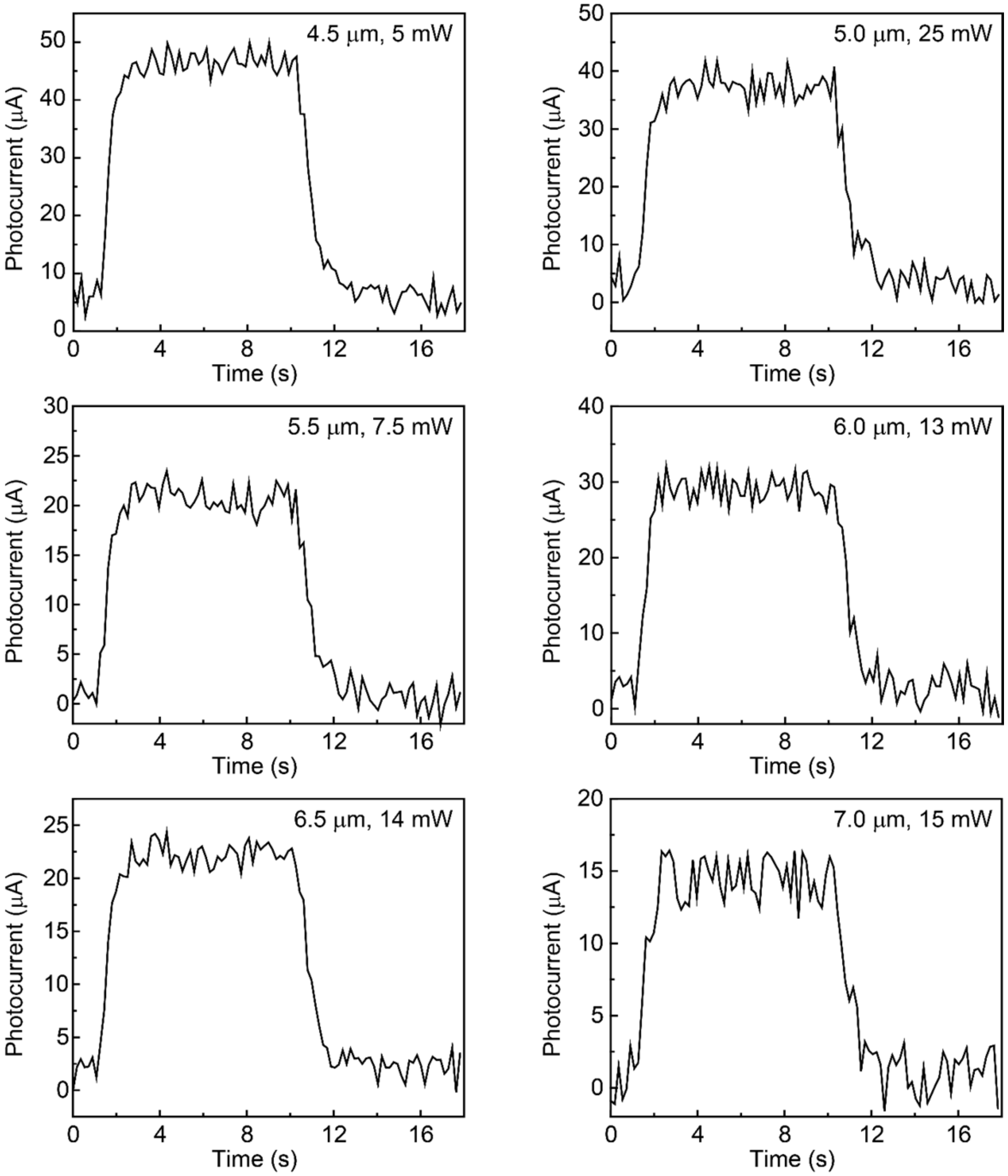

Supplementary Figure 5. Time-dependent photocurrent measurements under mid-infrared laser illumination with wavelengths ranging from 4.0 μm to 7.0 μm. The diameter of the laser spot is 3 mm.

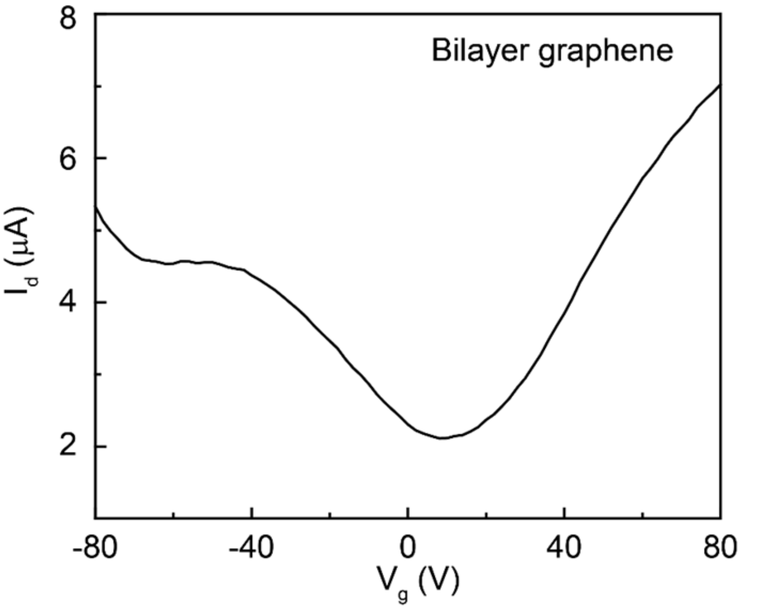

Supplementary Figure 6. I_d-V_g curve of bilayer graphene FET measured under ambient conditions.

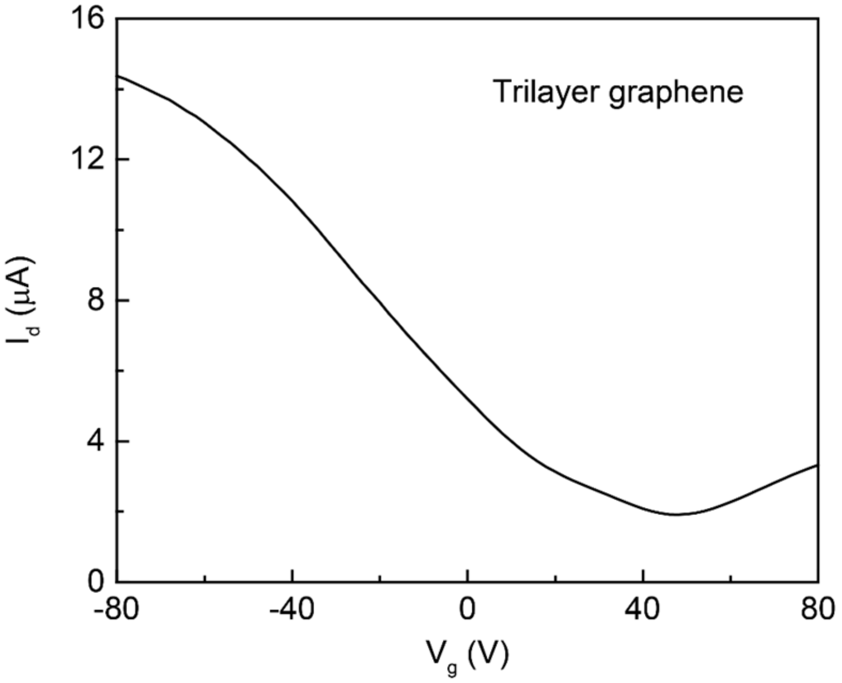

Supplementary Figure 7. I_d-V_g curve of tri-layer graphene FET measured under ambient conditions.

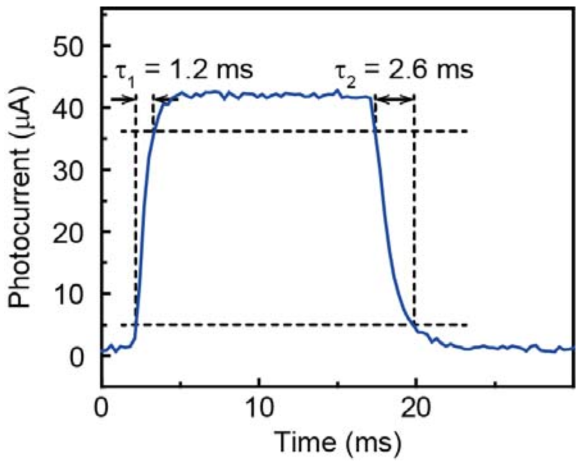

Supplementary Figure 8. Temporal photocurrent of the hybrid graphene/Ti_2O_3 photodetector measured under 10 μm QCL laser illumination with monolayer graphene.

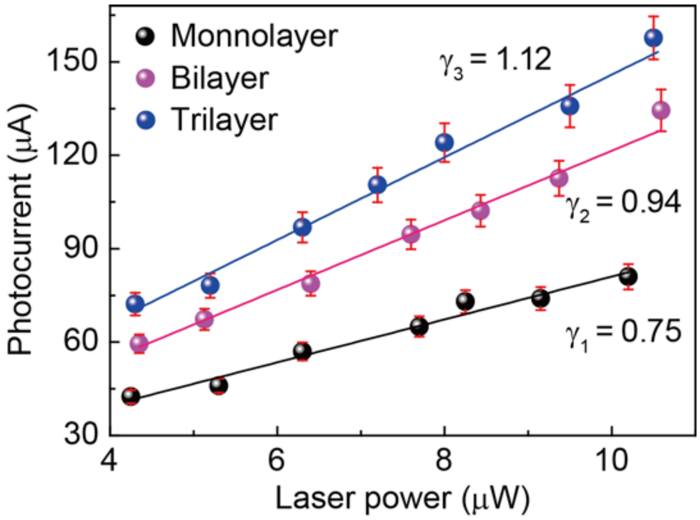

Supplementary Figure 9. Laser power dependences of the photocurrent measured with hybrid graphene/Ti_2O_3 photodetectors with different layers of graphene. Error bars represent standard deviation.

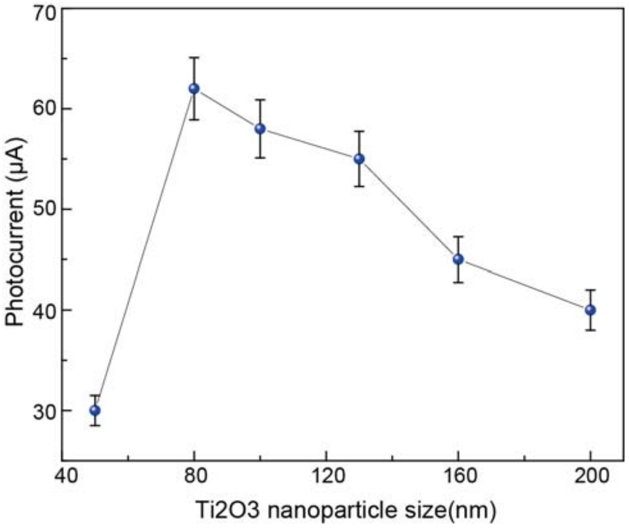

Supplementary Figure 10. Size dependence of the photocurrent of the hybrid graphene/Ti_2O_3 photodetectors. Error bars represent standard deviation.

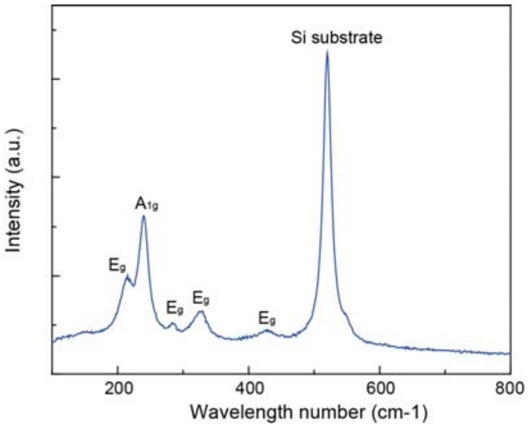

Supplementary Figure 11. Raman spectrum of thin film Ti_2O_3 grown on graphene.

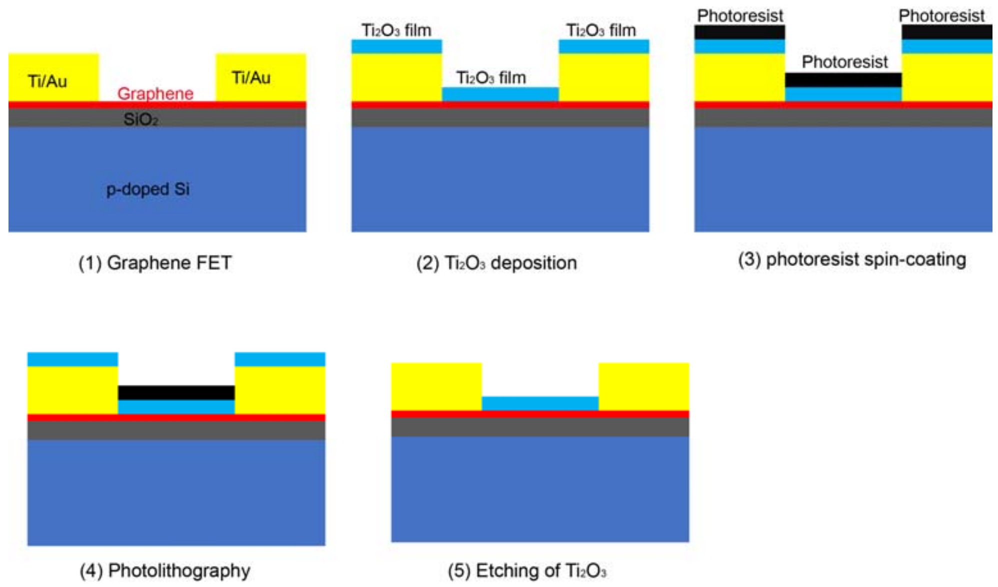

Supplementary Figure 12. Experimental illustration of fabrication of Ti_2O_3 thin film on graphene FET.

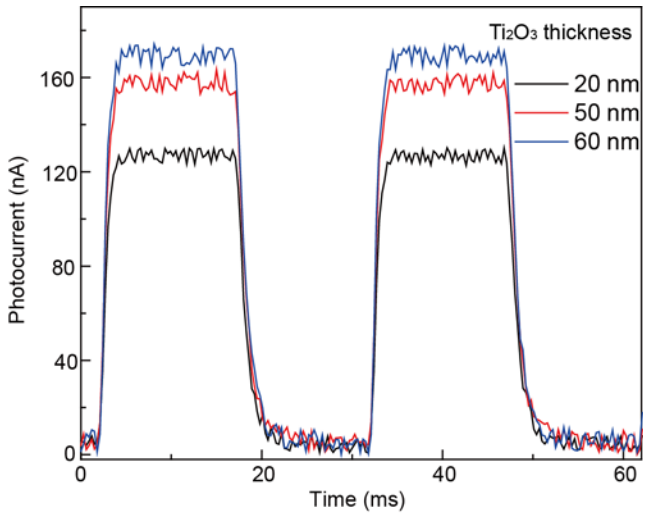

Supplementary Figure 13. Photocurrent of three samples with different Ti_2O_3 thicknesses.

Supplementary methods

Material characterization: The SEM images are measured using the field emission scanning electron microscopy (FESEM, JEOL JSM-6340F). The high-resolution TEM and electron diffraction pattern images are taken on JEOL JEM-2010. The crystallographic phases of the sample are determined by Bruker D8 ADVANCE diffractometer, equipped with the Cu Kα radiation source and LynxEye detector. Raman measurements are carried out with a confocal micro-Raman system (WITec alpha300) at ambient conditions, using a diode laser (excitation wavelength is 532 nm). Before the measurement, the system is calibrated with Si substrate with the Raman peak located at 520 cm^{-1} and the laser power is kept by less than 0.5 mW to avoid laser induced damage of the graphene sample. TEM images and EDS spectrum are collected by an FEI TitanST commercial TEM, operated at 300 V. The XPS spectra are carried out by using a Thermo Kalpha spectrometer with monochromated Al K alpha radiation, a dual beam charge compensation system and a constant pass energy of 50 eV. The adventitious C1s peak set to 285.00 eV is employed for signal calibration.

Light absorption. The absorption spectra for Ti_2O_3 nanoparticles are measured using the Varian carry 6000i spectrophotometer and FTIR. The spectra, collected from 200 nm to 1400 nm, are measured in the transmission mode when the nanoparticles are dispersed in ethanol, using the 6000i spectrophotometer. The background from quartz cuvette and ethanol has been deducted. The absorption spectra of Ti_2O_3 nanoparticles are measured in the transmission mode by FTIR, where the nanoparticles are dropped on a KBr substrate. The background from KBr substrate is deducted. The measurements are all performed in the ambient conditions.

First-principle calculations. The electronic structure and DOS are studied by the hybrid function in view of the HSE exchange[1-4], which are performed based on the projector augmented wave approach in the Vienna ab initio simulation package[5]. The Hubbard U_{eff} (=U-J) is imposed on Ti *3d* orbitals with the Dudarev implementation[6]. The crystal structure of electronic self-consistent interactions is optimized by using a plane-wave cut-off energy of 450 eV, and a Γ–centered Monkhorst-Pack k-point mesh of 5×5×5.

FET device fabrication process: Atomic-layer graphene flakes are mechanically exfoliated from a crystal of highly oriented pyrolytic graphite (HOPG) using adhesive 3M-tape and deposited on a silicon wafer with a 285-nm thermalized SiO_2 layer. The location and quality of graphene are identified by optical contrast using an optical microscope and Raman spectroscopy. Then, graphene-based FETs with a heavily doped silicon substrate as the back-gate electrode are fabricated by standard photolithography and e-beam evaporation. The optical images of the devices are shown in Figure S3 to Figure S5. Consequently, the Ti_2O_3 nanoparticles are dispersed in ethanol solution (~5%, wt %) and spin-coated on the surface of the graphene channel (4000 rpm). The devices are then dried in an oven under 80 °C for 12 hours.

Supplementary Note 1

Characterization: The SEM images are measured using the field emission scanning electron microscopy (FESEM, JEOL JSM-6340F). The high-resolution TEM and electron diffraction pattern images are taken on JEOL JEM-2010. The crystallographic phases of the sample are determined by Bruker D8 ADVANCE diffractometer, equipped with the Cu Kα radiation source and LynxEye detector. Raman measurements are carried out with a confocal micro-Raman system (WITec alpha300) at ambient conditions, using a diode laser (excitation wavelength is 532 nm). Before the measurement, the system is calibrated with Si substrate with the Raman peak located at 520 cm^{-1} and the laser power is kept by less than 0.5 *mW* to avoid laser induced damage of the graphene sample. TEM images and EDS spectrum are collected by an FEI $Titan^{ST}$ commercial TEM, operated at 300 V. The XPS spectra are carried out by using a Thermo Kalpha spectrometer with monochromated Al K alpha radiation, a dual beam charge compensation system and a constant pass energy of 50 eV. The adventitious C1s peak set to 285.00 eV is employed for signal calibration.

Light absorption. The absorption spectra for Ti_2O_3 nanoparticles are measured using the Varian carry 6000i spectrophotometer and FTIR. The spectra, collected from 200 nm to 1400 nm, are measured in the transmission mode when the nanoparticles are dispersed in ethanol, using the 6000i spectrophotometer. The background from quartz cuvette and ethanol has been deducted. The absorption spectra of Ti_2O_3 nanoparticles are measured in the transmission mode by FTIR, where the nanoparticles are dropped on a KBr substrate. The background from KBr substrate is deducted. The measurements are all performed in the ambient conditions.

First-principle calculations. The electronic structure and DOS are studied by the hybrid function in view of the HSE exchange[1-4], which are performed based on the projector augmented wave approach in the Vienna ab initio simulation package[5]. The Hubbard U_{eff} (=U-J) is imposed on Ti *3d* orbitals with the Dudarev implementation[6]. The crystal structure of electronic self-consistent interactions is optimized by using a plane-wave cut-off energy of 450 eV, and a Γ–centered Monkhorst-Pack k-point mesh of 5×5×5.

FET device fabrication process: Atomic-layer graphene flakes are mechanically exfoliated from a crystal of highly oriented pyrolytic graphite (HOPG) using adhesive 3M-tape and deposited on a silicon wafer with a 285-nm thermalized SiO_2 layer. The location and quality of graphene are identified by optical contrast using an optical microscope and Raman spectroscopy. Then, graphene-based FETs with a heavily doped silicon substrate as the back-gate electrode are fabricated by standard photolithography and e-beam evaporation. The optical images of the devices are shown in Fig.S3 to Fig. S5. Consequently, the Ti_2O_3 nanoparticles are dispersed in ethanol solution (~5%, wt %) and spin-coated on the surface of the graphene channel (4000 rpm). The devices are then dried in an oven under 80 °C for 12 hours.

Supplementary Note 2

Electrical and optoelectronic measurement: The electrical characteristics were examined by a semiconductor analyzer (Agilent, B1500A). The photoresponsivity measurement was performed in a digital deep level transient spectroscopy (BIORAD) system with mid-infrared lasers (Tunable CW/Pulsed External Cavity Quantum Cascade Laser, Daylight Solutions from 4.0 µm to 7 µm and 9.75 µm to10.48 µm) to illuminate the whole devices. The detectivity are calculated based on the following expressions[7]:

$$D = \frac{I_{ph}}{\mathrm{P}\sqrt{2\mathrm{e}I_{dark}}} \qquad \text{(Supplementary Equation 1)}$$

where I_{ph} and I_{dark} are the photocurrent dark current, P is the illumination power, e is the electron charge. The experimental setup is shown as Supplementary Figure 4. In a brief description, the emission wavelength of the quantum cascade laser (QCL) in the Mid-IR is controlled by the laser controller and is modulated by mechanical optical chopper, then the laser emission is focused to the sample area by a mid-IR lens. The electrical signal is collected by a semiconductor measurement system (Agilent 1500A) and analyzed by lock-in amplifier (SR830). We also added the experimental setup in the revised manuscript and supplementary information. Noise spectra are acquired by a spectrum analyzer (Keysight M9018A) with biased supplied by Agilent 1500A at ambient conditions.

Supplementary Note 3

The charge transfer from a semiconducting absorber to an adjacent two-dimensional material is layer-dependent[8]. In our experiment, as clearly shown in Figure 4e, the photoresponse of the hybrid graphene/Ti_2O_3 photodetector presents a noteworthy enhancement when the graphene channel increases from monolayer to trilayer, which is opposite to the observations in PbS QD sensitized MoS_2 atomic layers[9]. The layer-dependent photoresponse is further verified by the power dependence of the photocurrents, as shown in Supplementary Figure 9. The photocurrent increases with the incident laser power, which satisfies the relation: $I_{pc} = CP^{\gamma}$, where C is a constant and P is the illumination laser power, respectively. We obtain the fitting parameter γ_1 = 0.78 for the hybrid monolayer graphene/Ti_2O_3 photodetector, while γ_2 = 0.94 and γ_3 = 1.12 for the bilayer case and trilayer cases, respectively. Generally, the γ value indicates the recombination kinetics of photocarriers by the trap states in both the carrier injection interface and the transport channels[10]. As a result, the increase of the γ value with the increase of graphene layers can be attributed to the enhancement of the transfer rate of the photoexcited holes from Ti_2O_3 to the underneath graphene. The layer-dependent charge transfer efficiency accounts for the competition between screening and absorption of the electric field of the dipoles of semiconducting Ti_2O_3 nanoparticles and the graphene layer[11-13].

Supplementary Note 4

The obtained the Ti_2O_3 nanoparticles are dispersed in ethanol assisted by ultrasonic treatment. Then the Ti_2O_3 nanoparticles are deposited on the graphene FET by the spin-coating method with the same spinning speed. As a result, we obtain hybrid nanoparticle Ti_2O_3/graphene devices with different particles sizes. The photoresponse of the devices are measurement with the same conditions with 10 μm QLC laser as the description in Supplementary Note 2. From the photocurrent evaluation as shown in Supplementary Figure 10, it is indicated that the photocurrent increases with the decrease of the size of Ti_2O_3 nanoparticles from 200 nm to 80 nm, however, the photocurrent decreases rigidly when the size decreases to 50 nm. The size dependent photocurrent can be attributed to the trade-off between charge separation efficiency and light absorption efficiency.

For larger size Ti_2O_3 nanoparticles, the number of nanoparticles in the FET channel is less. Furthermore, as the size of Ti_2O_3 nanoparticles is larger than the charge carrier diffusion length ($\boldsymbol{L_D}$), thus the charge transfer is inefficient. We need to mention that we are not able to measure the exact value of L_D because the absorption peak of Ti_2O_3 nanoparticles is in the mid-infrared regime as shown in Figure 1g. For smaller size Ti_2O_3 nanoparticles, the number of particles on the graphene channel is more and thus enable a higher light absorption. More importantly, if the nanoparticle size is near or less than the carrier diffusion length, the charge transfer would be more efficient than that in larger nanoparticles. This is verified by the increase of photocurrent while decreasing the nanoparticle size from 200 nm to 80 nm as shown in Supplementary Figure 10. However, further decrease of the nanoparticle size may introduce surface defects, which increase the scattering loss and thus decrease the photocurrent. From our experiments, we conclude that the optimized size of Ti_2O_3

nanoparticles is around 80 nm which enables both high light absorption and high charge transfer efficiency.

Supplementary Note 5

Thin film Ti_2O_3 grown on graphene and its photoresponse

Before the thin film deposition, graphene FETs with multiple electrodes are fabricated using standard photolithography and e-beam deposition processes. Thin film Ti_2O_3 are grown on graphene FET using pulsed laser deposition (PLD) method as shown in our previous works[14, 15]. The Ti_2O_3 powders are compressed into a pellet-shaped target and put in a high-vacuum chamber. The thickness of the thin film Ti_2O_3 can be controlled by the deposition time. The Raman spectrum of thin film Ti_2O_3 is shown in the Supplementary Figure 11, confirming the growth of corundum phase Ti_2O_3. After thin film Ti_2O_3 deposition, the Ti_2O_3 on the electrode area was etched away by HCl solution. The fabrication processes are shown as in Supplementary Figure 12.

The photodetectors based on thin film Ti_2O_3 grown on graphene are measured with the same conditions with 10 μm QLC laser as the description in Supplementary Note 2. The photocurrent of three samples with different Ti_2O_3 thicknesses is shown as in Figure 13. The results indicate that thin film Ti_2O_3/graphene hybrid photodetectors have a much lower photoresponse (~200 times lower) compared to these of nanoparticle hybrid graphene/Ti_2O_3 photodetectors. This can be explained by the following three factors. First, thin film Ti_2O_3 has less confinement in the lateral dimensions, as a result, the charge transfer efficiency in the vertical direction is less efficient because the carriers are favor of lateral diffusion. Second, the Raman spectrum indicates that thin film Ti_2O_3 has poor crystallinity because the Raman peaks are broader compared to these of Ti_2O_3 nanoparticles. As a result, the defect concentration and carrier scattering loss in thin film Ti_2O_3 is higher. Third, thin film Ti_2O_3 need to be grown be a high-temperature PLD method, graphene channel might

degrade during the growing process and thus decrease the performance of the photodetectors.

References

1. Perdew, J. P.; Burke, K.&Ernzerhof, M. Generalized Gradient Approximation Made Simple. *Phys. Rev. Lett.* **77**, 3865-3868 (1996)
2. Danov, K. D.; Petsev, D. N.; Denkov, N. D.&Borwankar, R. Erratum: Pair interaction energy between deformable drops and bubbles [J. Chem. Phys. 99, 7179 (1993)]. *J. Chem. Phys.* **100**, 6104-6104 (1994)
3. Delley, B. An all‐electron numerical method for solving the local density functional for polyatomic molecules. *J. Chem. Phys.* **92**, 508-517 (1990)
4. Head, J. D.&Zerner, M. C. A Broyden—Fletcher—Goldfarb—Shanno optimization procedure for molecular geometries. *Chem. Phys. Lett.* **122**, 264-270 (1985)
5. Kresse, G.&Furthmüller, J. Efficient iterative schemes for ab initio total-energy calculations using a plane-wave basis set. *Phys. Rev. B* **54**, 11169-11186 (1996)
6. Dudarev, S. L.; Botton, G. A.; Savrasov, S. Y.; Humphreys, C. J.&Sutton, A. P. Electron-energy-loss spectra and the structural stability of nickel oxide: An LSDA+U study. *Phys. Rev. B* **57**, 1505-1509 (1998)
7. Mak, K. F.&Shan, J. Photonics and optoelectronics of 2D semiconductor transition metal dichalcogenides. *Nat. Photon.* **10**, 216-226 (2016)
8. Gaudreau, L.; Tielrooij, K. J.; Prawiroatmodjo, G. E. D. K.; Osmond, J.; de Abajo, F. J. G.&Koppens, F. H. L. Universal Distance-Scaling of Nonradiative Energy Transfer to Graphene. *Nano Lett.* **13**, 2030-2035 (2013)
9. Kufer, D.; Nikitskiy, I.; Lasanta, T.; Navickaite, G.; Koppens, F. H. L.&Konstantatos, G. Hybrid 2D–0D MoS_2–PbS Quantum Dot Photodetectors. *Adv. Mater.* **27**, 176-180 (2015)
10. Xia, F.; Mueller, T.; Lin, Y.-m.; Valdes-Garcia, A.&Avouris, P. Ultrafast graphene photodetector. *Nat. Nanotechnol.* **4**, 839-843 (2009)
11. Raja, A.; Montoya−Castillo, A.; Zultak, J.; Zhang, X.-X.; Ye, Z.; Roquelet, C.; Chenet, D. A.; van der Zande, A. M.; Huang, P.; Jockusch, S.; Hone, J.; Reichman, D. R.; Brus, L. E.&Heinz, T. F. Energy Transfer from Quantum Dots to Graphene and MoS_2: The Role of Absorption and Screening in Two-Dimensional Materials. *Nano Lett.* **16**, 2328-2333 (2016)
12. Zang, H.; Routh, P. K.; Huang, Y.; Chen, J.-S.; Sutter, E.; Sutter, P.&Cotlet, M. Nonradiative Energy Transfer from Individual CdSe/ZnS Quantum Dots to Single-Layer and Few-Layer Tin Disulfide. *ACS Nano* **10**, 4790-4796 (2016)
13. Tomadin, A.; Hornett, S. M.; Wang, H. I.; Alexeev, E. M.; Candini, A.; Coletti, C.; Turchinovich, D.; Kläui, M.; Bonn, M.; Koppens, F. H. L.; Hendry, E.; Polini, M.&Tielrooij, K.-J. The ultrafast dynamics and conductivity of photoexcited graphene at different Fermi energies. *Sci. Adv.* **4**, eaar5313 (2018)
14. Li, Y.; Weng, Y.; Yin, X.; Yu, X.; Kumar, S. R. S.; Wehbe, N.; Wu, H.; Alshareef, H. N.; Pennycook, S. J.; Breese, M. B. H.; Chen, J.; Dong, S.&Wu,

T. Orthorhombic Ti_2O_3: A Polymorph-Dependent Narrow-Bandgap Ferromagnetic Oxide. *Adv. Funct. Mater.* **28**, 1705657 (2018)

15. Li, Y.; Wang, Q.; An, M.; Li, K.; Wehbe, N.; Zhang, Q.; Dong, S.&Wu, T. Nanoscale Chemical and Valence Evolution at the Metal/Oxide Interface: A Case Study of Ti/$SrTiO_3$. *Adv. Mater. Interfaces* **3**, 1600201 (2016)
